# *Lactobacillus gasseri* JM1 Isolated from Infant Feces Alleviates Colitis in Mice via Protecting the Intestinal Barrier

**DOI:** 10.3390/nu15010139

**Published:** 2022-12-28

**Authors:** Shasha Cheng, Hongxuan Li, Yan Huang, Yue Su, Yu Li, Ai Jia, Yujun Jiang, Yu Zhang, Chaoxin Man

**Affiliations:** Key Laboratory of Dairy Science, Ministry of Education, College of Food Science, Northeast Agricultural University, Harbin 150030, China

**Keywords:** *Lactobacillus gasseri* JM1, intestinal barrier, gut microbiota, intestinal mucus, inflammatory bowel disease, ulcerative colitis

## Abstract

Ulcerative colitis (UC) is a chronic and recurrent inflammatory bowel disease, and the intestinal barrier is an important line of defense against intestinal disease. Herein, we investigated the effect of *Lactobacillus gasseri* JM1 at different doses (1 × 10^6^, 1 × 10^7^, 1 × 10^8^ CFU/day) on colitis mice and explored the possible mechanism. The results showed that *L. gasseri* JM1 alleviated DSS-induced colitis in mice, with reductions in disease activity index (DAI), histological scores and myeloperoxidase activity as well as alleviation of colonic shortening. Furthermore, *L. gasseri* JM1 regulated the levels of inflammatory cytokines TNF-α, IL-6, IL-1β, and IL-10; restored the expression of Claudin-3, Occludin, ZO-1, and MUC2; and increased the number of goblet cells and acidic mucin. The 16S rDNA sequencing results indicated that intervention with *L. gasseri* JM1 balanced the gut microbiota structure by elevating the abundance of beneficial bacteria (*Oscillospira*, *Clostridium* and *Ruminococcus*) and decreasing that of harmful bacteria (*Shigella* and *Turicibacter*). Meanwhile, the contents of short-chain fatty acids (SCFAs) increased. In conclusion, *L. gasseri* JM1 could alleviate intestinal barrier damage in colitis mice by modulating the tight junction structures, intestinal mucus layer, inflammatory cytokines, gut microbiota, and SCFAs. It can be considered a potential preventive strategy to alleviate colitis injury.

## 1. Introduction

The gastrointestinal tract (GIT) is not only an important organ for digestion and absorption of nutrients, but it also protects the host from toxins and pathogens, thus maintaining environmental homeostasis. In addition, as the first line of defense against external threats, the intestinal barrier effectively separates the luminal contents from the host tissues, which is essential for the health of animals and humans [1,2]. It consists of a mucus layer secreted by goblet cells, a single layer of epithelial cells, and a mucosal lymphatic system containing complex immune cells [3]. When the intestinal barrier is damaged, it may lead to changes in intestinal permeability, causing intestinal diseases, such as inflammatory bowel disease (IBD), irritable bowel syndrome (IBS), food allergies, and obesity [4]. IBD is a disease of unknown origin characterized by chronic inflammation of the GIT, including Crohn’s disease (CD) and ulcerative colitis (UC), with clinical manifestations of persistent diarrhea, usually accompanied by blood and mucus [5]. Although the pathogenesis of IBD is still unclear, accumulated data have suggested that the disease is the result of a combination of genetic and environmental factors that could alter the balance of the intestinal environment and cause intestinal barrier dysfunction [6]. It has been demonstrated that all the tight junction proteins, mucous layer, and gut microbiota were altered [2,7,8,9]. Drug treatments, including aminosalicylates and steroids, are still commonly used for IBD, but long-term administration may cause side effects. New treatments are being researched, such as probiotics, prebiotics, and some microbial metabolites [10,11].

Lactic acid bacteria (LAB), including several *Lactobacillus gasseri* (*L. gasseri*), are potential probiotics that can maintain gut homeostasis and contribute to the health of the host when given in sufficient amounts [12,13]. In addition, *L. gasseri* is one of the major species for early intestinal colonization, which is widely present in humans as a commensal bacterium [14]. Recent studies have also demonstrated that the use of LAB can alter the composition of gut microbiota, reduce intestinal damage, alleviate intestinal inflammation, ameliorate type 2 diabetes, and enhance immunomodulatory effects from in vitro and in vivo models [15,16,17,18,19]. Therefore, using probiotics to regulate gut microbiota is considered to be a way to promote health and treat certain diseases.

Recent evidence suggested that different probiotics have a direct or indirect positive effect on animals in experimental models of IBD. In different mouse models, dextran sulfate sodium (DSS)-induced colitis has been widely used in the study of IBD because of its simplicity and many similarities with UC patients. In vivo studies have shown that probiotics such as probiotic mixture VSL#3, *L. fermentum*, *L. reuteri*, and *L. rhamnosus* improved the intestinal barrier by alleviating intestinal inflammation, decreasing intestinal barrier permeability, and enhancing mucosal integrity in mice with colitis [1,7,20,21]. Meanwhile, *L. gasseri* was used to evaluate the protective effect of the intestinal barrier in colitis, and the results showed that the levels of the inflammatory cytokines were restored and mucosal damage was avoided [14,19]. Although many promising results have been achieved with probiotics in alleviating DSS-induced colitis, how probiotics affect the GIT and their underlying mechanisms are unclear and need to be further elucidated.

Previously, we conducted in vitro studies on the environmental tolerance, adhesion, and immunomodulatory activity of *L. gasseri* JM1 isolated from the feces of healthy infants. The results showed that *L. gasseri* JM1 could survive better in a high acid and high bile salt environment, better adhere to Caco-2 cells, and alleviate LPS-induced inflammation in Caco-2 cells [22]. Therefore, based on the previous study and combined with the current research on probiotics to improve the intestinal barrier, we proposed to continue to investigate the effects of *L. gasseri* JM1 on the regulation of intestinal mucosal damage, mucus layer characteristics, and gut microbiota in mice with colitis.

## 2. Materials and Methods

### 2.1. Bacterial Strains and Culture Conditions

*L. gasseri* JM1 (GenBank accession number CP044412-CP044414) was isolated from the feces of healthy infants and cultured in Man Rogosa Sharp broth (Qingdao Hope Bio-Technology Co., Ltd., Qingdao, China) at 37 °C under aerobic conditions for 18 h. The precipitate was collected by centrifugation at 5000× *g* for 5 min, resuspended in skim milk, and freeze-dried. Then, the viable count of *L. gasseri* JM1 after freeze drying was determined, and the freeze-dried power of *L. gasseri* JM1 was dissolved to 5 × 10^6^, 5 × 10^7^, and 5 × 10^8^ CFU/mL when applied to gavage.

### 2.2. Mouse Model and Experimental Design

**Figure 1:**
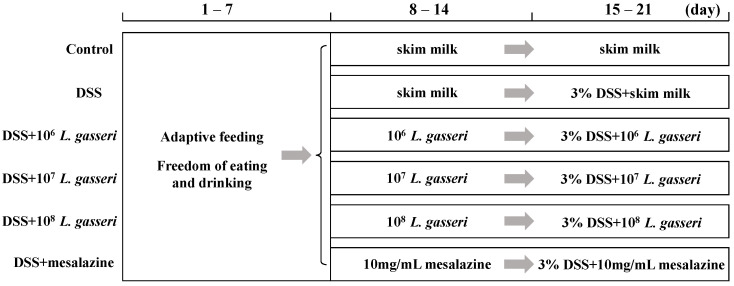
Grouping and intragastric administration in animal experiments.

The male C57BL/6 mice (6–7 weeks old, *n* = 48) were from Vital River Laboratory Animal Technology Co., Ltd. (Beijing, China). They were raised in cages at room temperature of 22 ± 2 °C and humidity of 55 ± 5% with a light/dark cycle of 12 h. Then, all mice were divided into six groups (*n* = 8 per group) and fed standard chow and sterile water. Figure 1 showed the experimental design with modifications to the previous description [23]. The mice had free access to water and food for the first week to acclimatize to the environment. Those in the control and DSS groups were intragastrically fed 200 uL of skim milk daily, and others were intragastrically fed 200 uL of *L. gasseri* with 5 × 10^6^, 5 × 10^7^, 5 × 10^8^ CFU/mL (1 × 10^6^, 1 × 10^7^, 1 × 10^8^ CFU/day) and 200 uL of mesalazine (10 mg/mL) that resuspended in skim milk for the second week. After 7 days, mice were administered 3% DSS (*w*/*v*, MP Biomedicals, LLC, Irvine, CA, USA) in their drinking water to induce colitis in all groups except the control mice. Meanwhile, mice in the control and DSS groups were intragastrically fed 200 uL of skim milk daily while mice in the *L. gasseri* and mesalazine groups were intragastrically fed 200 uL of *L. gasseri* with 5 × 10^6^, 5 × 10^7^, 5 × 10^8^ CFU/mL (1 × 10^6^, 1 × 10^7^, 1 × 10^8^ CFU/day) and 200 uL of mesalazine (10 mg/mL) for one week, respectively.

### 2.3. Histological Analysis for Colitis

During the DSS intervention, the changes in the body weight, fecal viscosity, and fecal occult blood were determined every day according to the previous studies [20,24]. The fecal occult blood of fecal samples from each mouse was determined by the fecal occult test kit (Nanjing Jiancheng Co., Ltd., Nanjing, China). Then, the disease activity index (DAI) was scored as previously described [25]. DAI = (Weight Loss Score + Fecal Traits Score+ Hidden Blood Score)/3. After the mice were sacrificed, serum and colon samples were collected. Colonic tissues were collected under sterile conditions and the entire colon length (from the cecum to the anus) was determined by a ruler. Next, the colonic tissues were placed in formalin for one day and then sectioned, and 5 mm thick sections were stained with hematoxylin-eosin (HE) and analyzed for histological features according to the previous method [26]. The remaining colon, serum, and fecal samples were collected for subsequent procedures.

### 2.4. Determination of Myeloperoxidase (MPO) Activity

The serum was obtained from mouse blood by centrifugation at 3000× *g* for 10 min. The level of MPO activity in serum was assessed by a commercial MPO assay kit (Nanjing Jiancheng Co., Ltd., Nanjing, China), and all procedures were performed by the manufacturer’s instructions.

### 2.5. Determination of Inflammatory Cytokines

To investigate the expression of targets, colonic tissues of mice and sterile phosphate buffer solution were placed in glass mills at a ratio of 1:9 and centrifuged at 3000× *g* for 20 min. The supernatants were collected to determine the concentrations of anti-inflammatory cytokines (IL-10) and proinflammatory cytokines (IL-1β, IL-6 and TNF-α) by commercial ELISA kits (Nanjing Jiancheng Co., Ltd., Nanjing, China).

### 2.6. Determination of Mucin and Goblet Cells

Fixed colonic tissues were embedded in paraffin and cut into 5 μm sections. The distribution of mucin in the colon was investigated by Alcian blue and periodic acid–Schiff (AB-PAS) staining as previously described with modifications [27]. Periodic acid–Schiff (PAS) staining was used to evaluate the number of goblet cells as previously described [28]. Specifically, the distribution of different mucus and the number of goblet cells were determined with Image-pro Plus 6.0 (Media Cybernetics, Inc., Rockville, MD, USA).

### 2.7. Reverse Transcription (RT) and Quantitative Real-Time PCR (qPCR)

To investigate the expression of tight junction protein-related genes (Occludin, Claudin-2, ZO-1, Claudin-3) and the MUC2 gene, the total RNA of every colonic tissue was isolated by Simple P Total RNA Extraction Kit (Bioer Technology Co., Ltd., Hangzhou, China) as previously described with modifications [29]. The concentration of the RNA and the value of A260/A280 were determined by QuickDrop (Molecular Devices Corporation, San Jose, CA, USA), and the quality was assessed by agarose gel electrophoresis. The cDNA was synthesized by PrimeScript™ RT Reagent Kit (TaKaRa Bio, Dalian, China) with modifications as previously described [29]. All procedures were performed by the manufacturer’s instructions. The mRNA levels were determined by the QuantStudio^®^ 3 Real-Time PCR system (Applied Biosystems, Foster City, CA, USA) with TB Green^®^ Premix Ex Taq™ II (TaKaRa Bio, Dalian, China). The specific RT-qPCR primers for target genes are shown in Table 1. The changes in gene levels were expressed as the fold of the control.

### 2.8. Gut Microbiota Sequencing and Analysis

The contents of cecum from mice were collected to assess the changes in the composition of gut microbiota. The total DNA was obtained using the most suitable extraction method. PCR amplification of the V3-V4 region of the 16S rRNA gene was performed using primers (F: ACTCCTACGGGAGGCAGCA R: GGACTACHVGGGTWTCTAAT) as described previously [30]. DNA samples were sequenced by Illumina MiSeq. Bacterial diversity analysis of the sequence data was conducted after sequencing.

### 2.9. Determination of Short-Chain Fatty Acids (SCFAs)

The contents of cecum (0.8000 ± 0.010 g) were accurately weighed, and 10% suspension was prepared. The suspension (500 μL) was added to crotonic acid metaphosphate solution (100 μL), frozen at −30 °C for 24 h, and then centrifuged at 8000 rpm for 3 min to remove impurities, such as protein. The supernatant was filtered by 0.22 μm water filters, and the contents of SCFAs were quantitatively analyzed by gas chromatography [31].

### 2.10. Statistical Methods

SPSS software was used for one-way ANOVA, and Excel and GraphPad Prism 8.02 were used for graph production. All experiments were repeated three times, and the results were expressed as mean ± standard deviation (Mean ± SD). *p* < 0.05 indicated that the results were significantly different.

## 3. Results

### 3.1. Effects of L. gasseri JM1 on Physiological Indicators

**Figure 2:**
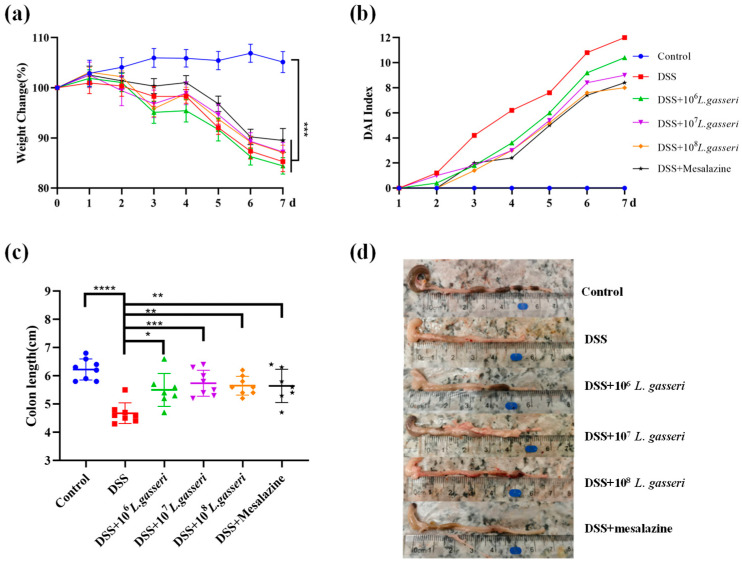
*L. gasseri* JM1 alleviated colitis in mice. (**a**) The change of body weight in mice, (**b**) the change in DAI index, and (**c**,**d**) the change in colon length. * *p* < 0.05, ** *p* < 0.01, *** *p* < 0.001, **** *p* < 0.0001 represent significant difference between groups.

DSS-induced colitis is the most widely used animal model of UC in the study [32]. The changes in body weight, DAI index, and colonic length were determined during the experiment, as shown in Figure 2. The control group showed a slight upward trend in body weight (Figure 2a), and the DAI index remained at 0 (Figure 2b). There were extremely significant differences in the rate of weight loss in other groups compared to the control group (*p* < 0.001). Nevertheless, *L. gasseri* JM1 (10^7^ and 10^8^) and mesalazine groups slowed down the weight loss in mice with colitis. The DAI index was the highest in the DSS group while the intervention with different concentrations of *L. gasseri* JM1 reduced the DAI index in a dose-dependent manner. The length of the colon is one of the important indicators for the evaluation of colitis [33]. The length of the colon was 6.23 ± 0.37 cm (Figure 2c) in the control group with normal reddish and granular feces (Figure 2d). In comparison, the length of the colon in DSS-induced mice was 4.68 ± 0.36 cm, accompanied by colonic edema, intestinal wall hemorrhage, and irregular stools. Intervention with *L. gasseri* JM1 and mesalazine could significantly prevent the shortening of the colon, which was consistent with the results of the DAI index.

### 3.2. Effects of L. gasseri JM1 on the Histological Analysis of Colitis

**Figure 3:**
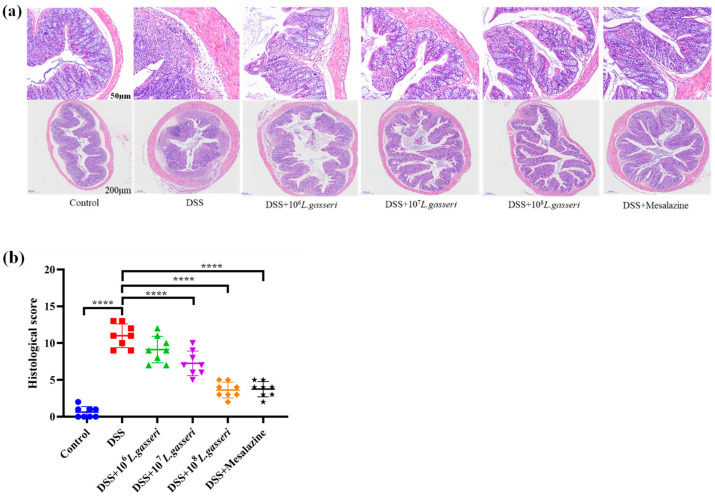
HE staining and histological scores of colitis mice. (**a**) The colonic microsections stained with HE and (**b**) histological score of colitis mice. **** *p* < 0.0001 represents a significant difference between groups.

After HE staining, pathological changes were observed under microscope and photographed. The tissues of mice were expressed as 50 μm and 200 μm (Figure 3a), and histological scores were evaluated (Figure 3b). The results indicated that the histological score of the control group was 0.625 ± 0.744, with intact epithelial cells in the mucosal layer, abundant intestinal glands, and many goblet cells, and no necrosis or inflammatory infiltration were seen. However, accompanied by diffuse infiltration of inflammatory cells, there was extensive necrosis in the intestinal mucosal layer with fibrous tissue hyperplasia and severe crypt injury in the DSS group. The histological score (11.00 ± 0.603) was significantly different from that of the control group (*p* < 0.01), which was consistent with the results of HE staining. *L. gasseri* JM1 groups with different concentrations ameliorated the mucosal injury of colonic tissues in mice to varying degrees in a dose-dependent manner. The most pronounced alleviation was observed in the 10^8^ *L. gasseri* JM1 group, accompanied by a large number of goblet cell proliferation, which was similar to the control group. Colon histological scores also confirmed these results. Therefore, *L. gasseri* JM1 could effectively alleviate tissue damage and maintain the integrity of the mucosal structure in colitis mice induced by DSS.

### 3.3. Effect of L. gasseri JM1 on Serum MPO Activity in Colitis

**Figure 4:**
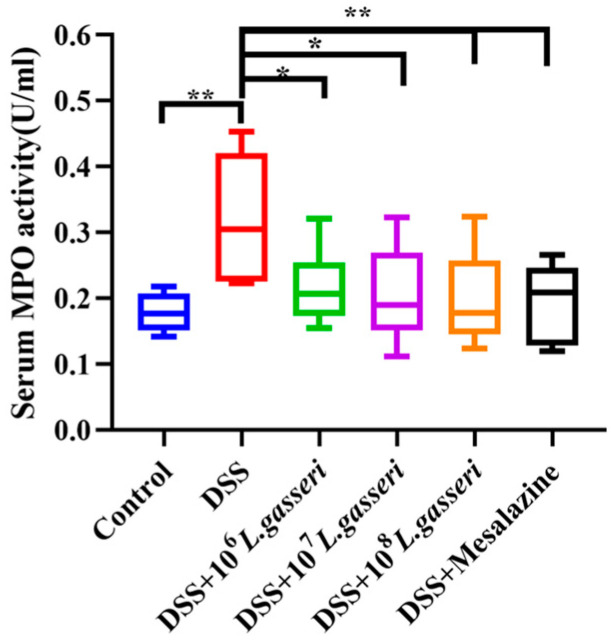
Changes of MPO activity in serum. * *p* < 0.05, ** *p* < 0.01 represent significant difference between groups.

Changes in MPO activity were related to the degree of neutrophil infiltration in the relevant tissues [34]. Therefore, MPO can be considered one of the essential indicators of inflammation in colitis. As shown in Figure 4, MPO activity in the DSS group (0.318 ± 0.09 U/mL) was significantly higher than that in the control group (0.179 ± 0.03 U/mL, *p* < 0.01). On the contrary, the MPO activity of the *L. gasseri* JM1 and mesalazine groups showed a downward trend with statistical significance compared with the DSS group (*p* < 0.05, *p* < 0.01), which was close to that of the control group. The results showed that *L. gasseri* JM1 and mesalazine could significantly reduce inflammation in mice with colitis by decreasing serum MPO activity to varying degrees.

### 3.4. Effect of L. gasseri JM1 on Inflammatory Cytokines Expression in Colitis

**Figure 5:**
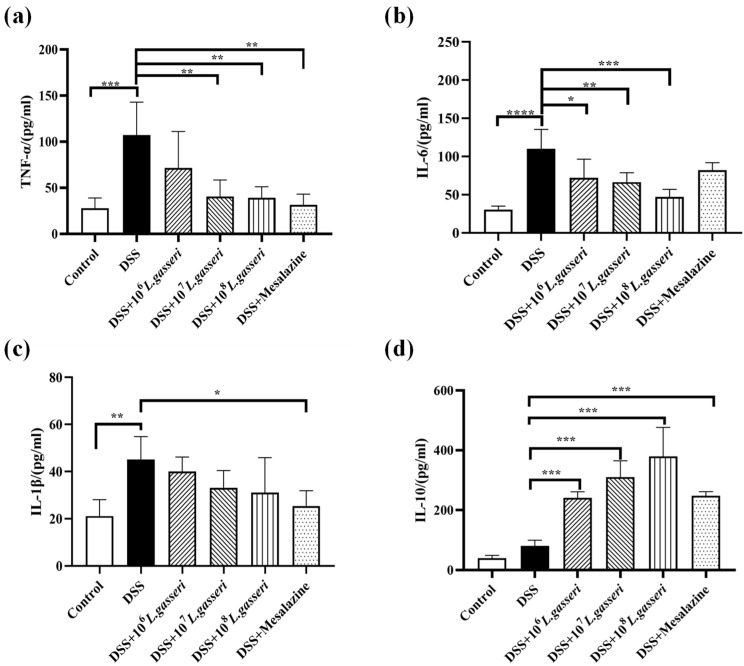
Changes of immune cytokines expression in colitis mice. (**a**) Levels of TNF-α, (**b**) levels of IL-6, (**c**) levels of IL-1β, and (**d**) levels of IL-10. * *p* < 0.05, ** *p* < 0.01, *** *p* < 0.001, **** *p* < 0.0001 represent significant difference between groups.

Disturbance of intestinal immune regulation is an essential factor in the pathogenesis of IBD, in which proinflammatory cytokines TNF-α, IL-6, IL-1β and anti-inflammatory cytokine IL-10 are the key indicators reflecting the degree of inflammation in the model of colitis [35]. As shown in Figure 5, the levels of TNF-α, IL-6, and IL-1β were increased compared with the control group (*p* < 0 0.001, *p* < 0.01) while the level of IL-10 was also increased in the DSS group, which may be related to autoimmune regulation of mice in response to external stimuli. Meanwhile, compared with the DSS group, the levels of TNF-α, IL-1β, and IL-6 decreased, and the level of IL-10 significantly increased (*p* < 0.001) after mesalazine intervention. In addition, the levels of TNF-α, IL-1β, and IL-6 in *L. gasseri* JM1 groups all decreased to varying degrees in a dose-dependent manner with significant upregulation of IL-10 (*p* < 0.001). The results showed that mesalazine and *L. gasseri* JM1 could regulate inflammatory cytokines, thereby alleviating intestinal inflammation in mice.

### 3.5. Effect of L. gasseri JM1 on the Staining of the Colonic Mucus Layer and Goblet Cells in Colitis

**Figure 6:**
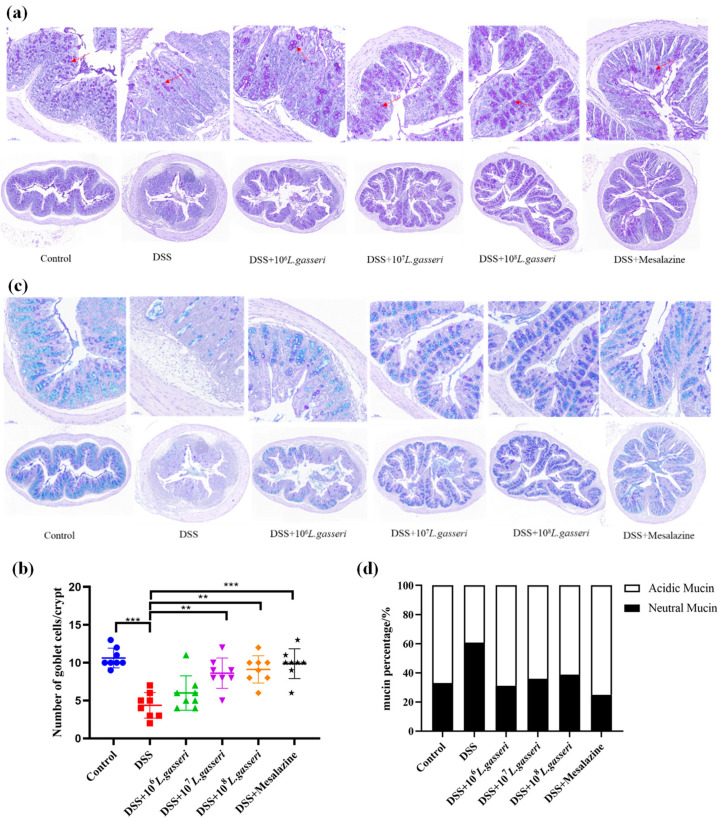
Staining and distribution of goblet cells and mucin. (**a**) Staining of goblet cells in colonic tissue, (**b**) counting of goblet cells in colonic tissue, (**c**) staining of mucin and distribution in colonic tissue, and (**d**) distribution of mucin in colonic tissue. ** *p* < 0.01, *** *p* < 0.001 represent significant difference between groups.

Colonic goblet cells, usually located in the mucosal epithelium, are typical mucus-secreting cells that continuously secrete mucus and play an essential role in the intestinal barrier. Microscopic observation of mouse colonic tissue after PAS staining (Figure 6a) and counting of goblet cells (Figure 6b) showed that goblet cells and the structure of the mucus layer in the DSS group were significantly damaged or almost destroyed. Specifically, the number of goblet cells decreased to 4.38 ± 1.69/glandular fossa with an extremely significant downward trend compared with the control group (10.63 ± 1.30/glandular fossa, *p* < 0.001). Compared with the DSS group, the number of goblet cells significantly increased in the mesalazine group, reaching 9.88 ± 2.00/glandular fossa (*p* < 0.001). *L. gasseri* JM1 intervention significantly increased the number of goblet cells dose-dependently. Especially in the high-dose group (10^8^ *L. gasseri* JM1 group), *L. gasseri* JM1 intervention significantly improved the destruction of the mouse mucus layer in mice with an orderly arrangement of goblet cells and an increased coverage of intestinal mucus.

AB-PAS can show neutral mucin and acidic mucin of intestinal mucus better. Specifically, neutral mucin appeared red or purplish-red, and acidic mucin appeared blue while mixed mucin appeared blue–purple or violet–blue after staining [36,37]. As shown in Figure 6c–d, AB-PAS staining results of the control group were blue and purple, predominantly blue, and the percentage of acidic mucin reached 66.88%, indicating that the intestinal mucin layer was rich in mucin and mainly acidic mucin. On the contrary, the secretion of both neutral and acidic mucin was significantly reduced in the DSS group with the percentage of acidic mucin decreasing to 39.13% and the percentage of neutral mucin increasing to 60.87%. However, intestinal mucus secretion was restored, and acidic mucus secretion significantly increased in the mesalazine and *L. gasseri* JM1 groups.

### 3.6. Effect of L. gasseri JM1 on the Expression of Tight Junction Protein-Related Genes and MUC2 Gene in Colitis

**Figure 7:**
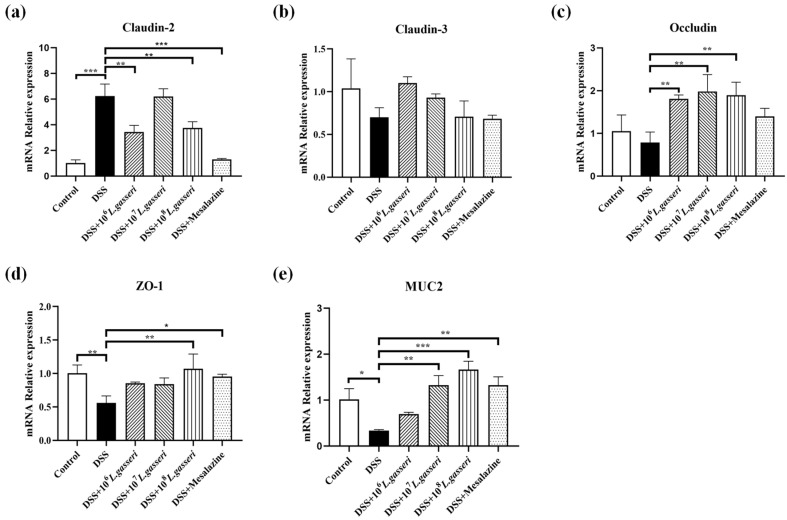
Regulatory effect of *L. gasseri* JM1 on tight junction protein-related genes and MUC2 gene in colitis mice. (**a**) mRNA relative expression of Claudin-2, (**b**) mRNA relative expression of Claudin-3, (**c**) mRNA relative expression of Occludin, (**d**) mRNA relative expression of ZO-1, and (**e**) mRNA relative expression of MUC2. * *p* < 0.05, ** *p* < 0.01, *** *p* < 0.001 represent significant difference between groups.

The epithelial barrier structure in DSS-induced colitis mice usually shows disruption of tight junction proteins and changes in intestinal permeability, increasing the probability of antigen invasion in the intestinal lumen [38]. Therefore, it is particularly crucial to determine the effect of *L. gasseri* JM1 on the mRNA expressions of intestinal tight junction proteins (Claudin-2, Claudin-3, Occludin and ZO-1) in mice with colitis. The results (Figure 7a–d) showed that the gene expression of Claudin-2 increased significantly (*p* < 0.001) while those of ZO-1, Claudin-3, and Occludin decreased to some extent in the DSS group. However, these trends were reversed after mesalazine intervention, similar to the control group. In addition, compared with the DSS group, the mRNA expression levels of ZO-1 and Occludin increased and that of Claudin-2 decreased, but that of Claudin-3 showed an upward trend without significant difference in *L. gasseri* groups. These results suggested that different doses of *L. gasseri* JM1 regulated the transcriptional level of tight junction protein-related genes.

The main component of mucus was MUC2. As shown in Figure 7e, the expression of the MUC2 gene significantly decreased in the DSS group compared with the control group (*p* < 0.05), which was consistent with the staining results and the reduction of goblet cells. However, compared with the DSS group, the expression of the MUC2 gene significantly increased after mesalazine, 10^7^, and 10^8^ *L. gasseri* JM1 interventions, and it increased in the 10^6^ *L. gasseri* JM1 group without statistical difference. These results indicated that different doses of *L. gasseri* JM1 increased mucus secretion by increasing the transcriptional level of MUC2 in dose dependence and played a role in protecting the mucus barrier in mice with colitis.

### 3.7. Effect of L. gasseri JM1 on the Composition of Gut Microbiota in Colitis

**Figure 8:**
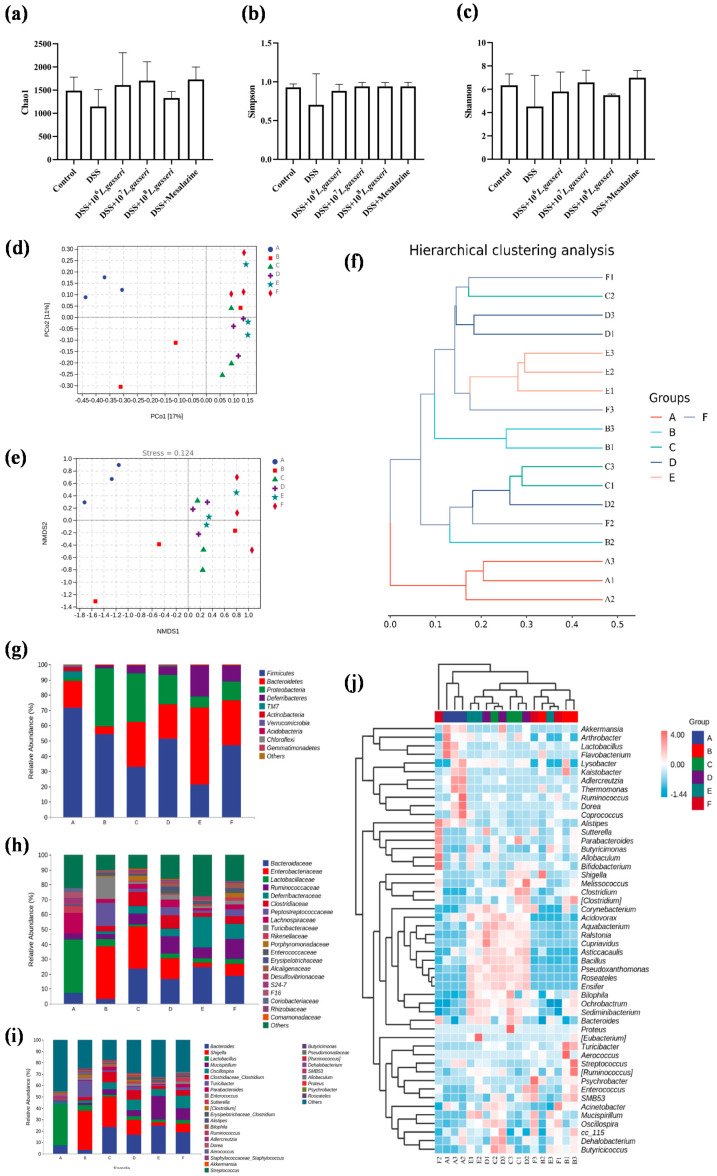
Differences in intestinal microbiota composition among the six groups (*n* = 3). (**a**) Chao1 index, (**b**) Simpson Index, (**c**) Shannon Index, (**d**) PCoA analysis of groups, (**e**) NMDS analysis of groups, (**f**) UPGMA hierarchical clustering analysis of microbiology in groups, (**g**) microbial composition at the phylum level of the cecal contents, (**h**) microbial composition at the family level of the cecal contents, (**i**) microbial composition at the genus level of the cecal contents, and (**j**) heatmap of genus composition combined with cluster analysis at the genus level. A stands for control group, B for DSS group, C for DSS + 10^6^ *L. gasseri* JM1 group, D for DSS + 10^7^ *L. gasseri* JM1 group, E for DSS + 10^8^ *L. gasseri* JM1 group, and F for DSS + mesalazine group.

Changes in gut microbiota are also indispensable factors in the pathogenesis of colitis. We analyzed whether the alleviating effect of *L. gasseri* JM1 in mice with colitis could be related to the improvement of gut microbiota. Alpha diversity analysis (Figure 8a–c) showed an increase in community composition after the intervention of *L. gasseri* JM1 and mesalazine, indicating an increase in microbial diversity and homogeneity. Beta diversity analysis was assessed (Figure 8d–f), including principal coordinates analysis (PCoA), nonmetric multidimensional scaling (NDMS) analysis, and hierarchical clustering analysis using the unweighted pair-group method with arithmetic means (UPGMA). PCoA and NDMS showed the distance and affinity of microbial communities and evaluated the differences in microbial communities between samples. UPGMA hierarchical clustering analysis presented the similarity of microbial communities between samples in the form of hierarchical trees. These showed that the community structure of samples in the control group and the 10^8^ *L. gasseri* JM1 group were clustered respectively while those of the samples in the DSS group were dispersed, confirming that DSS could change the diversity and stability of intestinal microbiota in colitis mice, and it gradually improved after the intervention of *L. gasseri* JM1 at different doses.

As shown in Figure 8g, the gut microbiota of mice in different groups was analyzed at the phylum level. It could be seen that the composition of gut microbiota in the six groups was similar, but the abundance of microorganisms was greatly different. Generally, the dominant gut microbiota at the phylum level was Firmicutes, Bacteroidetes, and Proteobacteria, accounting for about 80%. Compared with the control group, the abundance of Firmicutes (54.27% vs. 71.92%) and Bacteroidetes (5.19% vs. 17.04%) in the DSS group decreased while that of Proteobacteria (37.86% vs. 2.44%) increased. On the contrary, compared with the DSS group, the abundance of Proteobacteria decreased in the *L. gasseri* JM1 and mesalazine groups, and the abundance of Bacteroidetes increased, especially in the 10^8^ *L. gasseri* JM1 group. In addition, it is noteworthy that 10^7^ *L. gasseri* JM1 could maintain the abundance of Firmicutes in the intestinal tract (51.59% vs. 54.27%), and mesalazine intervention had a similar effect on the gut microbiota of mice with colitis as that of 10^7^ *L. gasseri* JM1 intervention. At the family level and genus level (Figure 8h–j), compared with the control group, the abundance of gut microbiota showed an increase in *Shigella*, *Turicibacter*, *Parabacteroides*, and *Staphylococcus* and a decrease in *Bacteroides*, *Lactobacillus*, *Alistipes*, *Ruminococcus*, *Dorea*, and *Akkermansia* in the DSS group. However, these trends were reversed after *L. gasseri* JM1 and mesalazine intervention. Among them, *Mucispirillum*, *Allobaculum*, and *Bifidobacterium* were enriched considerably in individual samples. In general, different samples from the control group clustered together, indicating that the community structure of gut microbiota in the control group was highly similar whereas that in the DSS group was severely damaged with specific differences. After the intervention of *L. gasseri* JM1, the gut microbiota structure also had a high similarity but was significantly different compared with the DSS group. Meanwhile, the community composition analysis at the genus level combined with cluster analysis further confirmed the results of beta diversity analysis.

### 3.8. Effect of L. gasseri JM1 on SCFAs in Colitis

**Figure 9:**
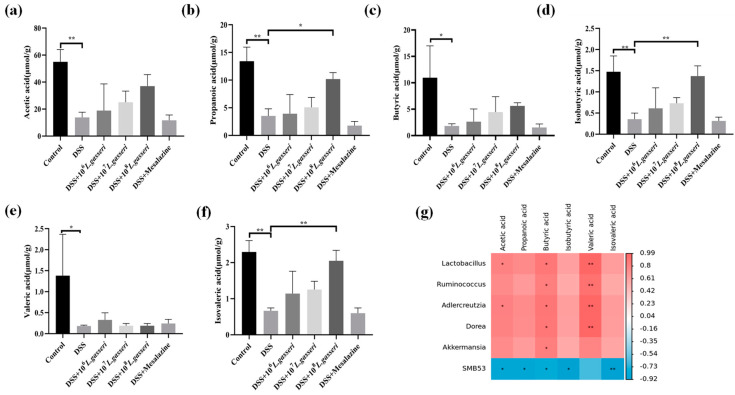
The effect of *L. gasseri* JM1 on SCFAs in mice with colitis. (**a**) Change of acetic acid content, (**b**) change of propionic acid content, (**c**) change of butyric acid content, (**d**) change of isobutyric acid content, (**e**) change of valeric acid content, (**f**) change of isovaleric acid content, and (**g**) correlation heatmap between SCFAs and relative abundance of differential microorganisms. * *p* < 0.05, ** *p* < 0.01 represent significant difference between groups.

Current studies have shown that the levels of SCFAs were reduced in mice with colitis, and some probiotics have been shown to regulate the production of intestinal SCFAs [39,40]. Therefore, we determined to explore the effect of *L. gasseri* JM1 on the contents of SCFAs in the cecum of mice. As shown in Figure 9a–f, the contents of SCFAs in the DSS group significantly decreased compared with the control group (*p* < 0.05). However, compared with the DSS group, the contents of all five SCFAs, except valeric acid, were increased in a dose-dependent manner after the intervention of *L. gasseri* JM1. Specifically, the contents of propionic acid, isobutyric acid and isovaleric acid significantly increased in the 10^8^ *L. gasseri* JM1 group (*p* < 0.05, *p* < 0.01), higher than those in the mesalazine group. Further, the correlation analysis between SCFAs and differential bacterial genus (Figure 9g) showed that acetic acid was positively correlated with *Lactobacillus* and *Adlercreutzia*; butyric acid and valeric acid were positively correlated with *Lactobacillus*, *Ruminococcus*, *Adlercreutzia*, and *Dorea*; and in addition, butyric acid was also positively correlated with *Akkermansia*. Simultaneously, the abundance of the above microbiota increased after the *L. gasseri* JM1 intervention. It could be seen that *L. gasseri* JM1 could exert its probiotic regulation effect when entering the intestinal tract, significantly regulating the intestinal metabolic function of mice with colitis and increasing the content of SCFAs by improving gut microbiota, among which, 10^8^ *L. gasseri* JM1 could play a more prominent role.

## 4. Discussion

In recent years, the increasing incidence of IBD has made it one of the critical diseases affecting human health. The use of microorganisms to regulate colitis has become a research hotspot. Many studies have shown that probiotics can alleviate colitis in animal models or clinical IBD patients to varying degrees by enhancing the mucosal barrier, regulating immune response, and improving the composition of gut microbiota [41,42,43]. The results of this study showed the effect of *L. gasseri* JM1 on the intestinal mucosal injury, intestinal barrier, and intestinal metabolites in UC mice and explored the mechanism of its alleviation of UC.

In this study, the acute UC model was induced with 3% DSS drinking water for one week. The DSS group began to show symptoms, such as weight loss, lethargy, loss of appetite and loose stools, on the second day of modeling followed by watery stools and gradual deterioration on the fifth day of model construction, indicating the successful establishment of the acute UC model. The degree of inflammation increased with the increase of modeling time, resulting in weight loss. However, the reason for the slight upward trend in body weight on the fourth day of modeling was tentatively speculated to be that the mice were also gavaged with *L. gasseri* JM1 at the initial stage of inflammation, and the inflammation continued to cause weight loss in mice, which gradually regained weight on the fourth day after adaptation [44]. No remission trend was observed in the 10^6^ *L. gasseri* JM1 group, presumably due to inadequate dosage of probiotics. The higher the DAI index, the higher the degree of inflammation. With probiotic and drug interventions, the DAI index increased more slowly than that in the DSS group, and the symptoms of loose stools and bloody stools were relatively reduced. In addition, the length of the colon was an important indicator related to the degree of inflammation. We found the length of the colon was recovered and edema was improved to some extent with the intervention of *L. gasseri* JM1 and mesalazine. In addition, Yang et al. found that the colonic length was recovered in DSS-induced colitis after the intervention of *Bifidobacterium breve* CCFM683 [26], which was similar to the result of this study. Similarly, the pathological analysis of the colon showed consistent results that *L. gasseri* JM1 reversed the degree of intestinal mucosal injury in mice, indicating it was more effective at higher doses.

Intestinal inflammation is the pathological manifestation of colitis caused by DSS. MPO has been shown to be a local mediator of tissue injury and resultant inflammation and can also enter the extracellular fluid to participate in circulation [34]. It was found that both *L. gasseri* JM1 and mesalazine could effectively reduce the activity of serum MPO, indicating that *L. gasseri* JM1 could reduce inflammatory cell infiltration and inflammatory response in the mucosa. MPO is also associated with oxidative stress levels, and the increase of it leads to oxidative stress in mice [45]. It was observed that the activity of MPO was decreased under the action of *L. gasseri* JM1, and oxidative stress in mice may be partially alleviated, but further studies are still needed. Mesalamine, a gold treatment for colitis, is also a free radical scavenger and antioxidant that is considered to be the most effective [46]. In this study, the effectiveness of mesalamine was also confirmed, and the comparison between *L. gasseri* JM1 and mesalamine indicated that *L. gasseri* JM1 had some alleviating effect.

The regulation of LAB in the host immune system has always been one of the hot topics of research. Immune factors, including proinflammatory, anti-inflammatory, and growth factors, have important relationships with inflammation. For example, TNF-α is a critical proinflammatory cytokine causing intestinal inflammation and in the central position of many inflammatory cytokines, which can increase the secretion of inflammatory cytokines such as IL-1β and IL-6, leading to aggravation of intestinal mucosal damage [47]. In turn, the increased secretion of IL-6 further stimulates multiple targeted cells (APCs, T cells) to enhance the inflammatory response, and IL-1β is highly expressed in the inflammatory state, which is positively correlated with the degree of inflammation [7,35]. However, conversely, IL-10 has anti-inflammatory properties by inhibiting pro-inflammatory cytokines and chemokines with negative feedback regulation, thus acting as a downregulator of the inflammatory response. However, it was noteworthy that although the expression level of IL-10 in the DSS group was not significantly different from that in the control group, it also showed an upward trend, which may be related to the regulation of the nonspecific immune system activated by intestinal inflammation [9]. Similarly, we also found that the levels of TNF-α, IL-1β, and IL-6 were effectively reduced, and the level of IL-10 increased by *L. gasseri* JM1 intervention, indicating that *L. gasseri* JM1 exerted its immunomodulatory function to stimulate the organism to generate a cascade response and produce anti-inflammatory cytokines, which could play an immunomodulatory role in DSS-induced colitis.

Intestinal barrier dysfunction with increased intestinal permeability is another characteristic symptom of IBD pathophysiology. We found similar results to those of Chen [23]. Specifically, goblet cells were stained purple by PAS staining, indicating that the intervention of *L. gasseri* JM1 significantly increased the number of goblet cells and the secretion of mucin, presumably because *L. gasseri* JM1 colonized and reproduced in the intestine, which stimulated the development of immune tissues and immune response to promote the growth of goblet cells. Furthermore, highly glycosylated mucins are classified into neutral and acidic mucins according to glycol-branched chain components [48]. Compared with neutral mucus, acidic mucins better protect against bacterial translocation because particularly sulfated mucins appear less degradable by bacterial glycosidases and host proteases [49]. *L. gasseri* JM1 increased the secretion of mucus and the proportion of acidic mucin, presumably due to the colonization and reproduction of the strain in the intestinal tract to exert a regulatory effect, resistance to the adhesion and migration of pathogenic bacteria, and recovery of the intestinal microenvironment. MUC2, the main component of colonic mucus, is predominantly expressed in a healthy colon. However, the expression of MUC2 was low in colitis due to the impaired goblet cells and reduced secretion of mucus [48,50]. Yang et al. found that the mRNA expression level of MUC2, which was significantly downregulated under DSS induction, could be restored to normal after the intervention of *Bifidobacterium* [26]. Correspondingly, *L. gasseri* JM1 and mesalamine improved the damage degree of goblet cells and increased the number of goblet cells, MUC2 gene expression, and mucin secretion as well, which ultimately promoted the formation of the tight mucus layer and thus alleviated colitis by resisting to the invasion of bacteria and other antigens. These were consistent with the staining results of PAS and AB-PAS.

Tight junctions between intestinal epithelial cells are the most important mode of attachment and are a highly diversified structure composed of transmembrane and cytoplasmic proteins [38]. The Claudins family of proteins is an essential component of tight junctions. Claudins are a quad transmembrane protein with functions as a “fence” and “barrier”, which form tight junction chains by polymerization within the plasma membrane and secondary assembly with Claudins attached to the cell to cross the extracellular space and produce paracellular closure [38,51]. Occludin was the first important transmembrane protein to be identified. If it is damaged, it would lead to an increase in the permeability of paracellular to macromolecules. In addition, ZO-1 plays a role in connecting multiple types of tight junction proteins to maintain the integrity of the tight junction complex. Dou et al. found that *L. casei* ATCC 393 and its metabolites increased the expression levels of occludin, ZO-1, and Claudin-1 [52], and Din et al. found *Bifidobacterium bifidum* ATCC 29,521 also could increase the expression levels of ZO-1, MUC-2, and Claudin-3 [53]. In addition, it has been shown that DSS-induced colitis in mice was accompanied by an increase in the pore-forming protein Claudin-2 [54]. We found few relevant studies with simultaneous changes in Claudin-2 and Claudin-3, and the results showed that the extremely significant increase of Claudin-2 in the DSS group might increase the intercellular pores while the decrease of Claudin-3 would reduce the intercellular sealing ability. After intervention with *L. gasseri* JM1 and mesalazine, this phenomenon was alleviated in mice with colitis, and the mRNA expression levels of both Occludin and ZO-1 were effectively increased, which prevented the destruction of the intestinal barrier to maintain barrier integrity.

It is well-known that physiological disturbances induced by IBD are bound to cause changes in gut microbiota diversity, composition, and structure [55]. It was found by detailed analysis of taxonomic composition that the abundance of Proteobacteria at the phylum level, *Enterobacteriaceae*, *Peptostreptococcaceae*, and *Turicibacteraceae* at the family level, and *Shigella* and *Turicibacter* at the genus level all increased significantly, which were the result of gut microbiota dysregulation after DSS. It was worth noting that the increased abundance of Proteobacteria, including many pathogenic bacteria, could exacerbate the microecological imbalances in the intestine [56]. The intestinal pathogen *Shigella* was a major factor in diarrhea in children under five years of age [57]. Studies found that the abundance of intestinal microorganisms *Shigella* was significantly higher in UC patients than in healthy individuals [58]. *Turicibacter* significantly increased in this study, which was consistent with Munyaka’s results [59]. However, there are few studies on *Turicibacter* at present, and its association with inflammation needs to be further investigated. When *L. gasseri* JM1 intervened, the abundance of *Bacteroides*, *Mucispirillum*, *Oscillospira*, *Clostridium*, *Parabacteroides*, *Alistipes*, and *Ruminococcus* increased on the basis of a reduction in the pathogenicity-associated flora mentioned above. Among them, *Clostridiaceae* belonging to Firmicutes is a kind of human-friendly bacteria, which are involved in various metabolic processes in the host. In particular, *Clostridium* has been shown in several studies to be a butyric acid-producing bacterium that can alleviate the condition of colitis in mice, induce Treg cell differentiation, and reduce inflammation [60]. It has been suggested that *Mucispirillum* was a resident bacterium in the intestinal mucosa and could also be associated with immunity, as manifested by the generation of Treg cells and IgA. However, there was still much controversy regarding the function of *Mucispirillum*, and follow-up studies would be needed. Moreover, Federica and others found that the content of *Oscillospira* was proportional to health, and the abundance of this genus decreased when the body was inflamed [61]. Additionally, *Ruminococcus*, which belongs to *Ruminococcaceae*, was a crucial group of gut microbiota in UC that could decompose cellulose, especially fermenting resistant starch to produce metabolites [62]. In this study, it was observed that the abundance of *Ruminococcus* increased, suggesting that it may play a role in alleviating inflammation through the production of SCFAs.

Microbial-derived SCFAs, which are metabolites of gut microbiota, are beneficial to the host and have important functional effects, such as maintaining the water–electrolyte balance, reducing intestinal infections, lowering pH, and improving intestinal function [63]. Combined with the analysis of gut microbiota, it was found that the contents of SCFAs in the intestine significantly reduced in the DSS group while they increased dose-dependently after the *L. gasseri* JM1 intervention. This may be due to the fact that *L. gasseri* JM1 exerted its probiotic regulatory effect in the intestine, regulating the composition of gut microbiota, decreasing the abundance of harmful bacteria, and increasing the abundance of acetic and propionic acid-producing *Bacteroides* and *Ruminococcus* as well as butyric acid-producing *Clostridium* and *Lactobacillus*. This result was consistent with Bian, but unfortunately, *F. prausnitzii*, the typical butyric acid-producing strain, was not found in this study, and further research is still necessary [64].

## 5. Conclusions

Taken together, intervention with *L. gasseri* JM1 alleviated impaired barrier structure by decreasing the expression of Claudin-2 and increasing the expression of Claudin-3, Occludin, and ZO-1. In addition, it also increased the number of goblet cells, improved the secretion of acidic mucin, and maintained the integrity of the mucus layer in mice with colitis. In addition, intervention with *L. gasseri* JM1 achieved anti-inflammatory effects by regulating pro- and anti-inflammatory cytokines. Meanwhile, it increased the abundance of short-chain fatty acid-producing strains, such as *Clostridium* and *Ruminococcus*, and decreased the abundance of harmful bacteria, such as Shigel*la* and *Turicibacter*. Further, the contents of acetic acid, propionic acid, and butyric acid increased after intervention with *L. gasseri* JM1. Overall, both 10^7^ and 10^8^ *L. gasseri* JM1 were effective in reducing the damage caused by DSS-induced colitis, with 10^8^ *L. gasseri* JM1 having a more pronounced effect. Thus, it showed that the intervention with *L. gasseri* JM1 could protect the intestinal barrier through a combination of multiple actions, thereby preventing organismal damage caused by DSS-induced UC. The present study provided a theoretical basis for the microbiology-based prophylaxis of UC and provided relevant ideas for the application of this strain in the prevention of UC.

## Figures and Tables

**Table 1:** The specific RT-qPCR primers for target genes.

**Type**	**Gene**	**Base Sequence (5′→3′) ^a^**
Reference gene	β-actin	F: CCACTGTCGAGTCGCGTCC R: GTCATCCATGGCGAACTGGTG
Tight junction protein	Occludin	F: CACACCTCGTCGCTAGTGC R: AGATAAGCGAACCTGCCGAGC
	ZO-1	F: GGAGATGTTTATGCGGACGG R: CCATTGCTGTGCTCTTAGCG
	Claudin-2	F: GGTTCCTGACAGCATGAAATTT R: GCCATCATAGTAGTTGGTACGA
	Claudin-3	F: TCGGCCAACACCATCATCAG R: CTCAGACGTAGTCCTTGCGG
Mucoprotein	MUC2	F: GCTGACGAGTGGTTGGTGAATG R: GATGAGGTGGCAGACAGGAGAC

^a^: F represented the forward sequence and R represented the reverse sequence.

## Data Availability

Data in the project are still being collected, but all data used in the study is available by contacting the authors.
